# Central nervous system and immune cells interactions in cancer: unveiling new therapeutic avenues

**DOI:** 10.3389/fimmu.2025.1528363

**Published:** 2025-02-28

**Authors:** Junkai Wen, Yue Li, Wanli Deng, Zhi Li

**Affiliations:** ^1^ Putuo Hospital, Shanghai University of Traditional Chinese Medicine, Shanghai, China; ^2^ Department of General Surgery, Hubei Provincial Clinical Research Center for Umbilical Cord Blood Hematopoietic Stem Cells, Taihe Hospital, Hubei University of Medicine, Shiyan, China

**Keywords:** central nervous system, immune cells, cancer, tumor microenvironment, therapeutic targets

## Abstract

Cancer remains a leading cause of mortality worldwide. Despite significant advancements in cancer research, our understanding of its complex developmental pathways remains inadequate. Recent research has clarified the intricate relationship between the central nervous system (CNS) and cancer, particularly how the CNS influences tumor growth and metastasis via regulating immune cell activity. The interactions between the central nervous system and immune cells regulate the tumor microenvironment via various signaling pathways, cytokines, neuropeptides, and neurotransmitters, while also incorporating processes that alter the tumor immunological landscape. Furthermore, therapeutic strategies targeting neuro-immune cell interactions, such as immune checkpoint inhibitors, alongside advanced technologies like brain-computer interfaces and nanodelivery systems, exhibit promise in improving treatment efficacy. This complex bidirectional regulatory network significantly affects tumor development, metastasis, patient immune status, and therapy responses. Therefore, understanding the mechanisms regulating CNS-immune cell interactions is crucial for developing innovative therapeutic strategies. This work consolidates advancements in CNS-immune cell interactions, evaluates their potential in cancer treatment strategies, and provides innovative insights for future research and therapeutic approaches.

## 1 Introduction

Cancer continues to be a predominant cause of global mortality, resulting in millions of fatalities annually (1). Notwithstanding considerable progress in cancer research, our comprehension of its intricate developmental mechanisms remains insufficient. The central nervous system (CNS) is considered a distinct microenvironment, effectively segregated from peripheral circulation cells by the blood-brain barrier (2). Recent research has elucidated the deep association between the CNS and cancer, encompassing cancer-related neurological symptoms and the complex interactions between CNS and tumor cells (3). Research indicates that CNS signaling and substances generated inside the CNS might impact tumor growth and metastasis, while tumors can also influence CNS function (4). The CNS regulates tumor growth and metastatic capability via its interactions with the immune system (5). The molecular processes governing the relationship between the CNS and cancer are intricate, encompassing several signaling pathways, cytokines, neuropeptides, and neurotransmitters that are essential to these interactions. Nonetheless, contemporary research encounters substantial obstacles. Experimental circumstances frequently fail to accurately mimic the *in vivo* environment, necessitating more specialized therapeutic strategies to properly tackle these interactions. Addressing these gaps in comprehension and thoroughly clarifying the processes via which the CNS and immune system influence cancer progression may reveal new biological pathways and treatment approaches. This may have substantial ramifications for the development of novel therapies and the enhancement of patient outcomes.

The association between the neurological system and malignancies was initially identified in 1897, when researchers observed several nerve fibers encircling different forms of malignant tumors, indicating the nervous system’s significant involvement in tumor progression. This established the groundwork for cancer neuroscience. In 1985, Batsakis et al. were the pioneers in documenting perineural invasion (PNI) in head and neck malignancies, a process wherein cancer infiltrates adjacent nerves, facilitating tumor dissemination and nerve proliferation (6, 7). Researchers have acknowledged the significance of PNI in solid tumors, discovering its correlation with heightened tumor aggressiveness and unfavorable survival results (8). Recent research have indicated that the invasive potential of tumors correlates with nerve density in prostate (9), colorectal (10), head and neck (11), breast (12), pancreatic (13), and gastric cancers (14). Recent research has enhanced our comprehension of the intricate interplay between the neurological system and malignant tumors. Examining the mechanics of these connections and elucidating the nervous system’s impact on the tumor microenvironment (TME) may facilitate the development of more efficacious anti-tumor medicines.

The interplay between the CNS and immune cells is pivotal in the development and progression of cancer. Proximal connections between immune cells and neuronal terminals that regulate immune organs enable the immune system to enlist local neurons to influence immune responses (15). In contrast, immune cells and mediators also modulate synaptic plasticity in the neurological system. Studies indicate that the CNS modulates immune cell activity via many methods, including neurotransmitter secretion, neuroendocrine signaling, and neuro-immune interactions. Neural signals can directly affect immune cell activity and indirectly alter the tumor microenvironment via regulating immune cell migration and function (16). Neurotransmitters such as norepinephrine, serotonin, and acetylcholine have distinct regulatory effects on immune cells in different tumor types. These effects not only modify cytokine release by immune cells but also influence their capacity to identify and eliminate tumor cells. Additionally, the CNS indirectly modulates general immune system activity by activating or inhibiting particular neuronal pathways. This regulation include neurotransmitters, neuroregulatory factors, and the modulation of essential components within the immune system. Research has underscored the essential function of interactions between the CNS and the immune system in the start, progression, and treatment responses of cancer (15–17). The interaction between the CNS and immune cells is complex and precisely regulated, encompassing multi-tiered control of brain signals and immune cell activity. These interactions influence tumor biology and therapy results, highlighting the central nervous system’s crucial role in cancer.

The importance of CNS-immune cell interactions in cancer research is widely acknowledged, as they influence tumor growth, metastasis, the patient’s immunological status, and therapeutic responses (16). The central nervous system regulates immune cell function through neurotransmitters and neuropeptides, while immune cell states reciprocally affect the nervous system, creating a complex bidirectional regulatory network. This interaction can alter the tumor immune milieu, consequently affecting therapy efficacy and patient prognosis. Abnormal nervous system activation can result in immunosuppression, diminishing the anti-tumor immune response, whereas immune cell activation may induce neuroinflammatory responses, thereby impacting the patient’s quality of life. Comprehending the mechanisms of this interaction is essential for formulating new clinical strategies, as accurate regulation of this crosstalk may yield innovative pathways for personalized therapy, including the enhancement of immunotherapy results or the mitigation of side effects, thereby improving patient outcomes. This study consolidates current progress in CNS-immune cell interactions and examines their prospective use in cancer therapy techniques and preclinical investigations. Through the examination of recent discoveries in this domain, we intend to offer novel insights for forthcoming research and therapeutic approaches, promoting advancement in the pertinent areas.

## 2 Role of the CNS-immune cell interactions in cancer

**Figure 1:**
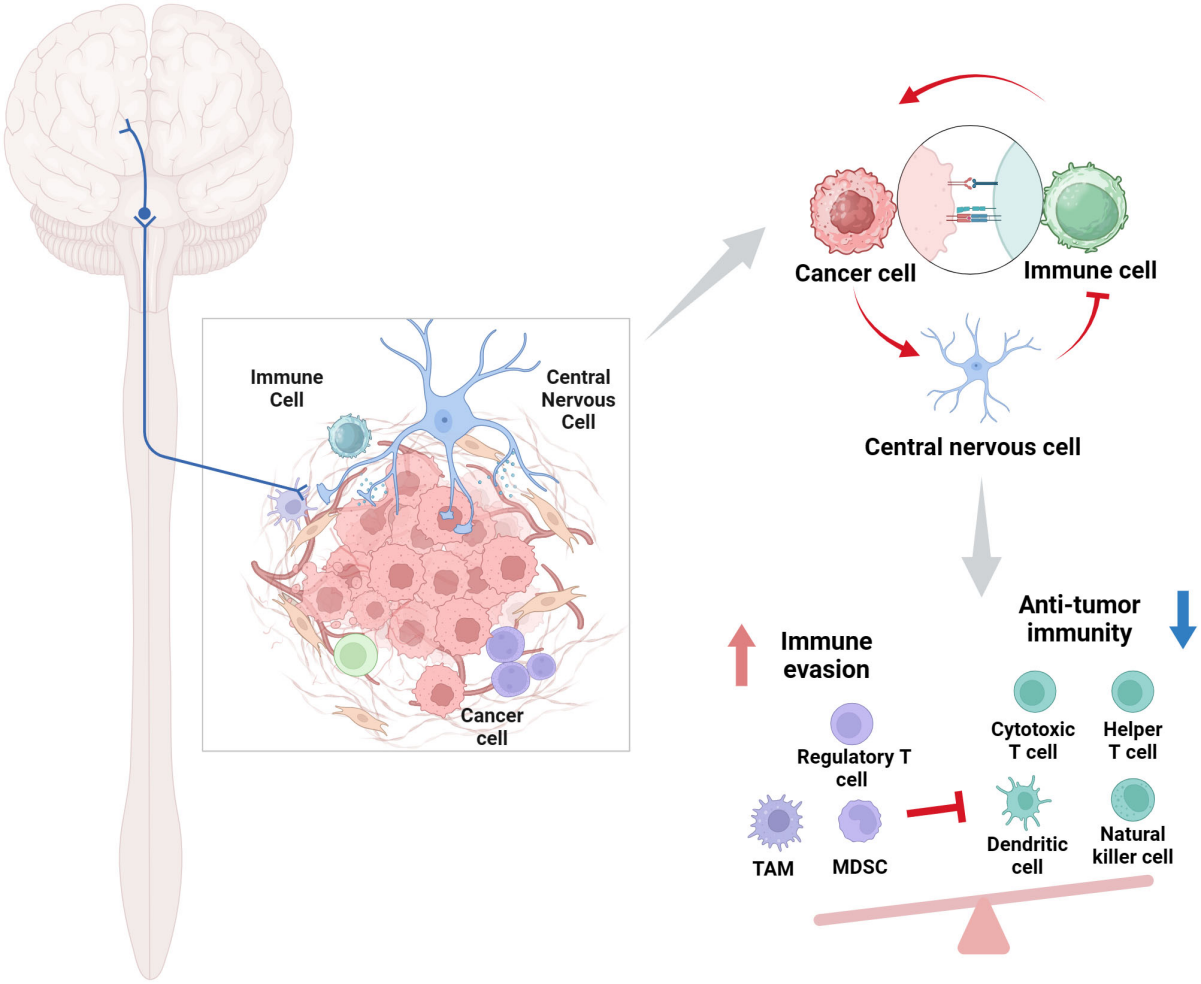
Role of the CNS-immune cell interactions in cancer. This intricate bidirectional regulatory network profoundly influences tumor progression, metastasis, patient immunological state, and treatment responses. Created with BioRender.com.

The neurological and immune systems collectively constitute an extensive physiological network tasked with overseeing and reacting to infections, inflammation, and malignancies (15). The notion of neuroimmune communication has been well-established, with numerous inflammatory symptoms arising from the impact of inflammatory mediators on the neurological system. Immune cells can synthesize neurotransmitters, acting as a non-neuronal source of these chemicals, with their release regulated by brain inputs or local tissue environmental signals (18). Upon exposure to tumor cells, the neurological system initiates a response, while neurotransmitters also affect the actions of immune cells. This complex interaction system allows us to efficiently address diverse immunological assaults, therefore preserving health and equilibrium (**Figure 1**).

### 2.1 The interaction modality between the CNS and the immune system

The interplay between the CNS and the immune system is pivotal in the advancement and progression of cancer. This interaction encompasses mechanisms including neurotransmitters, neuropeptides, inflammatory agents, and the hypothalamic-pituitary-adrenal (HPA) axis (19). The CNS directly regulates immune cell activity by the secretion of neurotransmitters and neuropeptides, including norepinephrine, which affects anti-tumor immunity. Norepinephrine can enhance PD-1 expression, modulate the activities of myeloid-derived suppressor cells (MDSCs) and macrophages, and inhibit anti-tumor immune responses (20). Moreover, immune cells can traverse the blood-brain barrier or infiltrate the cerebrospinal fluid, directly influencing neuronal cells and modulating neuronal survival and function during neuroinflammation (21). The reciprocal control between the CNS and immune cells transpires via the production of inflammatory agents and cytokines, which modify neuronal activity and influence immune cell migration, proliferation, and function. Glucocorticoids released by the HPA axis regulate systemic immune responses, affecting the magnitude and character of immunological reactions (22).

The CNS modulates immune cells, such as macrophages, within the TME in cancer, hence influencing tumor growth and spread. Upon detecting stress, the CNS activates the hypothalamic-pituitary-adrenal axis, converting this detection into a physiologic response. Activation of the HPA axis results in the secretion of neurotransmitters (e.g., glutamate and γ-aminobutyric acid), which subsequently affect peripheral effector systems (23). Certain neurotransmitters, like acetylcholine and monoamines, can have both excitatory and inhibitory effects, but neuropeptides such as epinephrine, norepinephrine, and dopamine are also involved in stress responses (24). The meninges, especially the dura mater, create a milieu conducive to the maturation of immature B cells, promoting negative selection via apoptosis to remove B cells that are reactive to the CNS (25). The choroid plexus serves as a central point for immune cell activity, governing the ingress of immune cells into the CNS and modulating leukocyte trans-epithelial migration through type II interferon signaling. T cells infiltrate the glial limitans into the CNS parenchyma, where they collaborate with resident myeloid cells, including perivascular macrophages, to execute immune surveillance and protective roles (26). Regulatory T cells can mitigate CNS inflammation by suppressing astrocyte activation, thus offering neuroprotection.

In the microenvironments of medulloblastoma and neuroblastoma, the interaction modalities between tumor cells and immune cells display both similarities and distinct differences. Tumor-associated macrophages (TAMs) serve as pivotal immune cells in the medulloblastoma microenvironment, exhibiting a dual role by promoting tumor growth and invasion through the secretion of growth factors, chemokines, and matrix-degrading enzymes, while also having the capacity to inhibit tumor growth via phagocytosis of tumor cells or cytokine secretion (27). This duality is closely associated with the leptomeningeal metastasis of medulloblastoma (28). In contrast, neuroblastoma’s TME is a complex ecosystem involving various immune cells and stromal cells, where TAMs, along with MDSCs, generally exhibit an immunosuppressive phenotype, counterbalancing the antitumor activities of NK cells and T cells (29). Notably, T cells, particularly CD8^+^ T cells, are critical in the medulloblastoma TME for their direct cytotoxicity against tumor cells, whereas their function is regulated by immune checkpoints like PD-1/PD-L1, allowing tumor cells to evade immune surveillance by expressing PD-L1 (27). Similarly, NK cells in both tumor types exert direct cytotoxic effects and secrete cytokines to initiate antitumor responses, yet their activity is compromised by the presence of immunosuppressive factors in the medulloblastoma TME (30). Dendritic cells play a role in activating T cells and initiating specific immune responses in both contexts and can be utilized in vaccine therapies to improve patient survival rates (31). The plasticity of neutrophils and the lesser-known function of B cells in the medulloblastoma TME, as well as the influence of interactions among immune cells on tumor progression, such as TAMs inhibiting T cell activity and the reciprocal activation of TAMs by T cells, further underscore the complexity of these interactions (27). In neuroblastoma, the TME’s influence on tumor cell phenotype, the role of extracellular vesicles in tumor homing and metastasis, and the immunosuppressive effects of components like NKG2D ligands highlight the nuanced differences in immune-tumor cell interactions between these two cancers (29).

### 2.2 Influence of the CNS on tumor growth and metastasis

The complex connections between immune cells, tumor cells, and neurons profoundly influence cancer progression (32). The creation, expansion, and advancement of tumors interfere with standard developmental and regenerative processes, establishing the nervous system as a crucial component in cancer pathophysiology. These connections include the impact of chronic stress on cancer, neurotransmitter regulation, neurogenesis, and the role of the nervous system in tumor growth and metastasis (33). Simultaneously, cancer and its therapies can influence and alter the neural system, establishing pathological feedback loops that result in neurological impairment and facilitate malignancy (34). These findings highlight the complexity and importance of neural participation in malignant tumor progression, providing insights for future therapeutic approaches aimed at the interactions between the nervous system and malignancies.

The supremacy of the nervous system over solid tumors and their microenvironments is crucial in tumor growth and metastasis (32). In female mice subjected to stress restriction, norepinephrine produced by the sympathetic nervous system triggers reactions at tumor locations (35). Tumor cells not only enhance neurogenesis but also aid the infiltration of autonomic nerve fibers into tumors, as evidenced in prostate cancer models. Chemical or surgical sympathetic nerve ablation, in conjunction with β2-adrenergic receptor gene deletion, can inhibit early tumorigenesis (36). Likewise, pharmaceutical inhibition or genetic alteration of M1 muscarinic receptors impedes tumor spread. The activation of adrenergic receptors in the neurological system can facilitate the development of several tumor types (37). The activation of the α1B adrenergic receptor can convert normal cells into tumor cells, whereas the activation of the β2 adrenergic receptor may cause DNA damage by inhibiting the tumor suppressor gene p53 and the protein kinase A (PKA) signaling pathway, thus promoting malignant transformation (38). Adrenergic signaling additionally increases the levels of matrix metalloproteinases (MMP) 2 and 9, hence augmenting the invasiveness of tumor cells. Inhibition of ADRB2 can reduce the expression of target genes, including MMP9, MMP2, and vascular endothelial growth factor (VEGF), via the cAMP-dependent PKA and Ras pathways, therefore diminishing tumor invasiveness. In ovarian cancer murine models, activation of ADRB2 elevates VEGF expression, augments angiogenesis, and stimulates tumor cell proliferation (39, 40). In murine models of gastric cancer, during the early stages of the disease, neural progenitor cells exhibit a tropism towards primary tumors and subsequently disperse to diverse locations. Specifically, doublecortin-expressing neural progenitor cells migrate from the neurogenic regions within the brain, traverse the blood-brain barrier, infiltrate the tumors, and ultimately differentiate into adrenergic neurons (41). Acetylcholine, by binding to M3 receptors, stimulates the release of nerve growth factor, facilitating neuroinvasion in stomach cancer (42). Cholinergic nerve fibers augment cellular immunity by diminishing PD-1 expression in T cells, thereby suppressing tumor proliferation (43). In breast cancer brain metastases, tumor cells express N-methyl-D-aspartate receptors (NMDAR), leveraging typical neuronal signaling pathways to promote proliferation and dissemination in the brain (39).

In the TME, direct interaction between neurons and glioma cells is essential for tumor proliferation and invasion. Glioma cells establish networks via gap junctions, facilitating potassium ion flow stimulated by neuronal activity, hence intensifying the impact of neurons on glioma cells. In adult glioblastoma models, synaptic connections promote the infiltration of tumor cells towards the periphery, similar to neuronal progenitor migration during normal brain development. Furthermore, within the TME, glioma cells enhance tumor growth by both establishing glutamatergic synapses and transducing signals mediated by AMPA receptor (44). Notably, glioma cells also release synaptogenic molecules, including thrombospondin-1 (TSP-1), which facilitate the functional reorganization of brain circuits (4). Electrophysiological investigations demonstrate that gliomas significantly respond to neuronal activity, leading to the reorganization of language networks. Brain-derived neurotrophic factor (BDNF) secreted by neurons amplifies current intensity in glioma cells, indicating dynamic connections between neurons and glioma cells (40). Additionally, glioma cells enhance potassium-induced current effects across gap junctions, promoting synchronized calcium transients within the neuron-glioma cell network. In glioblastoma, calcium signaling reliant on neuronal-glioma synapses facilitates tumor invasion, paralleling the pathways of neuronal progenitor migration in normal cortical development (45).

## 3 The interactions between CNS and immune cells in cancer

The CNS is crucial to the modulation of cancer immunity via many pathways. For instance, catecholaminergic neurons in the ventrolateral medulla specifically regulate CD8^+^ T cell activation to influence tumor progression (46). This suggests that the CNS can directly modulate antitumor immune responses through stress signaling pathways. Tumor cells can circumvent immune surveillance by emulating the anti-inflammatory processes of the CNS. Studies indicate that tumor cells contain CNS-specific N-acetyltransferase 8-like (NAT8L) and its metabolite N-acetylaspartate, which impede the cytotoxic activity of immune cells and interfere with the establishment of immunological synapses, thereby diminishing the immune system’s assault on tumors (3). This discovery underscores the essential function of CNS metabolites in tumor immune evasion.

**Figure 2:**
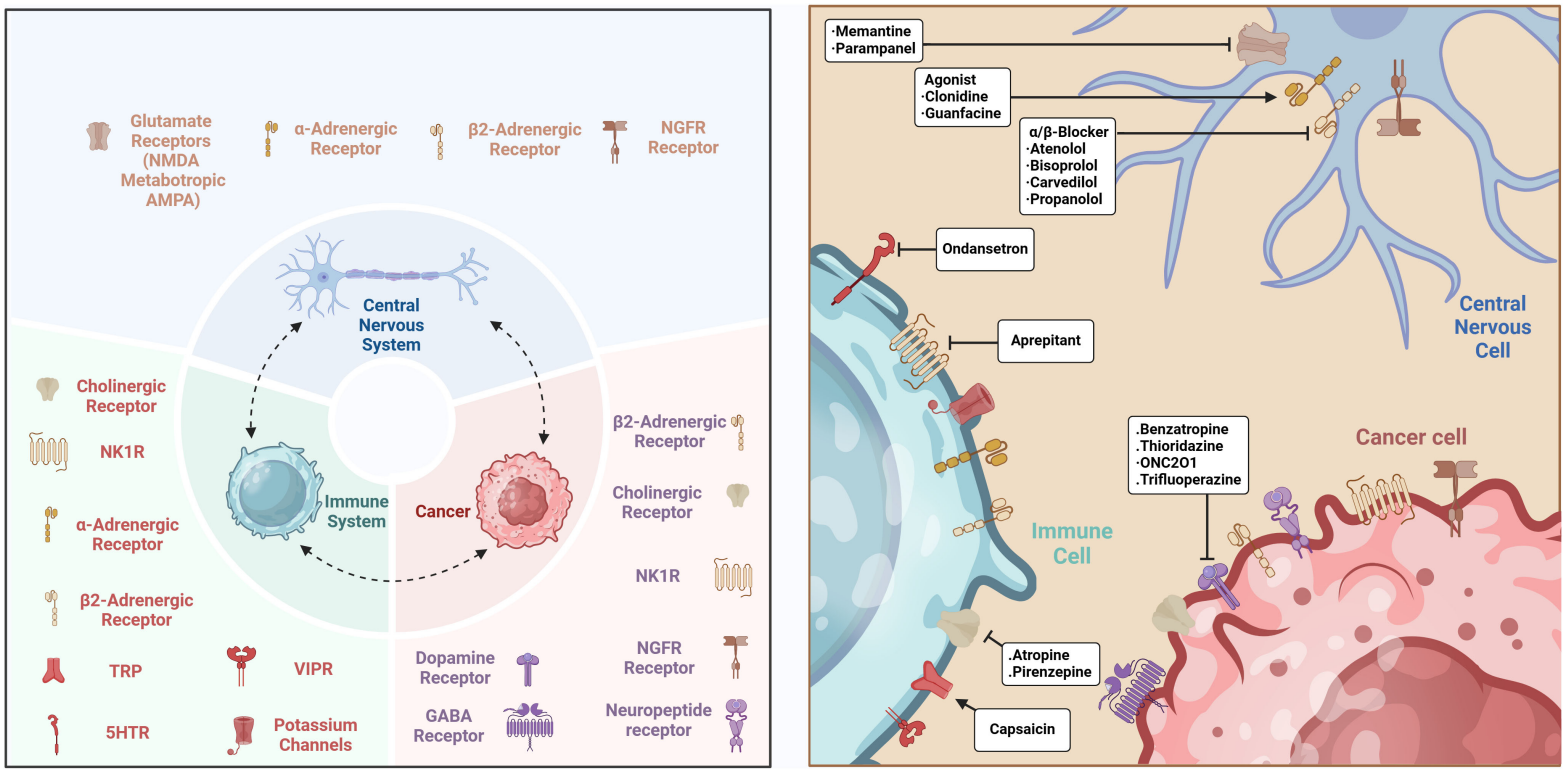
The interactions between CNS and immune cells in cancer. The connections between the CNS and immune cells govern the TME through many signaling pathways, cytokines, neuropeptides, and neurotransmitters, while also involving mechanisms that modify the tumor immune milieu. Created with BioRender.com.

Primary tumors can release neurotrophic factors, including members of the neurotrophic factor family or axon guidance molecules, to attract nerve fibers and enhance innervation. These brain signals directly impact tumor cells and indirectly promote tumor growth by altering the local immunological microenvironment. The CNS regulates the function of immune cells within the TME. Immune signals can affect neuroendocrine feedback loops, therefore modulating the activation and suppression of the immune system (47). This regulatory mechanism indicates that immune system modulation depends on both its inherent self-regulatory processes and external regulation by the CNS. The CNS interacts with various bodily regions via the autonomic nervous system and the neuroendocrine system, sustaining a dynamic equilibrium within the immune system that influences immune responses in the TME. Moreover, studies demonstrate that the nervous system might also affect cancer growth and therapy effectiveness by altering the intensity and characteristics of immune responses (**Figure 2**). These findings highlight the necessity of a comprehensive understanding of neuro-immune interactions as a critical element in enhancing cancer therapy options.

### 3.1 Bidirectional signaling between the CNS and the immune system in cancer

The CNS contains diverse immune cells, including microglia, B cells, and T cells from the periphery, which are essential for neurodevelopment, homeostasis, and neuroimmune responses (25). The CNS governs immune cell functions through many methods, affecting the self-renewal and differentiation capacity of neural stem cells (15). Throughout brain development, microglia engage in neurogenesis and myelination by phagocytosing cellular debris and secreting neurotrophic substances and reactive oxygen species, thus influencing neuronal survival (48, 49). Furthermore, they regulate synaptic pruning through the complement system and engage with neurons and astrocytes, influencing CNS function and homeostasis.

The viability and functionality of microglia are contingent upon the CSF1R signaling pathway, mediated by colony-stimulating factor 1 (CSF1) and IL-34, which profoundly affects the geographic specificity of microglial identity and function (48, 50). In inflammatory conditions, M1 pro-inflammatory microglia/macrophages impede the proliferation and differentiation of neural stem cells (NSCs) via inflammatory cytokines (e.g., IL-6 and TNF), while M2 anti-inflammatory microglia/macrophages facilitate NSC proliferation and neurogenesis by releasing anti-inflammatory cytokines (e.g., IL-10 and TGF-β) (51, 52). Moreover, M1 cells impede oligodendrocyte formation, whereas M2 cells promote it, presenting novel approaches for the repair of demyelinating injuries.

Under healthy conditions, T lymphocytes are limited in the CSF fluid yet modulate neuroinflammatory processes and the proliferation and differentiation of neural stem cells. Regulatory T cells, CD4^+^ Th2 cells, and CD4^+^ TMBP cells facilitate brain regeneration, but CD8^+^ T cells, CD4^+^ Th1 cells, and CD4^+^ Th17 cells impede it (26). B cells from peripheral blood develop into the B-1a subtype upon entering the CNS, where they markedly enhance the proliferation of oligodendrocyte precursor cells (OPCs) (25). Resident and invading B lymphocytes are essential for safeguarding the CNS and its barriers against infection. CXCL10 produced by astrocytes has been demonstrated to promote B cell ingress into the CNS (53). Nociceptive neurons are widely present in the splenic vasculature and B cell zones within the neuroimmune regulatory network, activating B cell CALCRL-RAMP1 receptors via CGRP, hence augmenting splenic germinal center responses and humoral immunity (54). Chronic psychological stress correlates with diminished NK cell activity and impaired lymphocyte proliferation, indicating that stress influences the immune system by modifying glucocorticoid and catecholamine levels, resulting in augmented humoral immunity but compromised cellular immunity (55, 56).

The nervous system significantly influences leukocyte migration and function via systemic modulation, affecting immune function in both healthy and stress-induced conditions (20, 57, 58). The activity of corticotropin-releasing hormone (CRH) neurons in the paraventricular nucleus stimulates the HPA axis, prompting the migration of lymphocytes and monocytes from peripheral organs to the bone marrow (19). Adrenergic transmission and sympathetic innervation govern the mobilization of hematopoietic stem cells into the circulation, a process especially evident during stress (59). Adrenergic signaling regulates immune cell movement within lymph nodes and modulates leukocyte gene expression by direct regulation of sensory neurons, thereby impacting total immunological functionality. In hippocampal neurons, TNF exposure elevates AMPA receptor levels, whereas treatment with soluble TNF receptor 1 yields a contrary effect (60). TNF also facilitates the endocytosis of GABAA receptors, hence diminishing inhibitory synaptic transmission. The expression of the B cell receptor CD22 is elevated in old microglia, and the inhibition of CD22 can reinstate phagocytic ability and enhance cognitive performance in elderly mice (61).

### 3.2 Neurotransmitter-immune cell interactions

The immunological environment of the CNS is distinctly specialized, with a separate lymphatic system and participating in intricate interactions with specialized immune cell populations in the cranial bone marrow (62). Numerous neuro-immune interactions occur in the brain’s border regions, potentially affecting the effectiveness of immunotherapy by altering immune cell activity (63). The interaction between neurotransmitters and immune cells demonstrates the significant influence of the neuro-immune system in governing the activities of both systems (64). Neurons can detect and respond to signaling molecules emitted by the immune system, whereas immune cells can also respond to neurotransmitters and other neuroregulatory chemicals (65). This bidirectional communication mechanism highlights the link between the neurological and immune systems, particularly in cancer, where the complex triangle interplay among neurons, immune cells, and cancer cells is more evident (66). This interaction not only influences the TME but also significantly regulates anti-tumor immune responses and affects the efficacy of immunotherapy (65, 67).

Norepinephrine, a crucial signaling chemical generated by the sympathetic nervous system, influences immunological modulation via binding to β2-adrenergic receptors on immune cells (45). This binding method initiates immunosuppressive responses, such as the elevation of PD-1 expression, modulation of MDSCs and macrophage activities, and enhancement of their migration to tumor locations (65). These effects collectively diminish the anti-tumor immune response, obstructing the immune system’s capacity to efficiently eradicate tumor cells. Norepinephrine also modulates vascular function via sympathetic innervation, restricting immune cell movement inside tissues and thereby reducing the immune system’s anti-tumor efficacy (68). Neurokinin CGRP, secreted by nociceptive sensory neurons, facilitates CD8^+^ T cell fatigue within the melanoma microenvironment, suggesting that the nervous system influences immunological responses through many routes, hence affecting immunotherapy outcomes (69).

The immune system can occasionally directly employ signaling molecules from neurons to modulate its actions (66). γ-Aminobutyric acid (GABA), a prevalent neurotransmitter, exerts inhibitory effects in the CNS and can also be produced by B lymphocytes (70). In a colon cancer murine model, GABA suppresses anti-tumor immune responses by interacting with GABAA receptors on CD8^+^ T cells, thereby facilitating tumor proliferation (71). GABA levels rise with tumor stage in original tumor specimens from patients with non-small cell lung cancer or colon cancer and exhibit a negative correlation with patient survival. Autocrine GABA signaling via GABAB receptors in colon cancer cells amplifies Wnt signaling and cellular proliferation, concurrently diminishing the expression and release of chemokines CCL4 and CCL5, which results in reduced infiltration of T lymphocytes and dendritic cells into tumors (72). The targeted elimination of GABA production in B cells markedly diminishes colon cancer proliferation, highlighting the essential function of neurotransmitters in influencing the tumor immunological milieu.

Serotonin (5-hydroxytryptamine) has been well examined for its regulatory functions in the CNS, but its significance in immunological regulation is also considerable (73). Serotonin produced by platelets can affect inflammatory processes and cellular growth. In orthotopic xenograft mouse models of gastric and pancreatic cancers, platelet-derived serotonin enhances PD-L1 expression on cancer cells via transglutaminase 2 (TGM2)-mediated serotonylation of histones, consequently inhibiting the immune system’s anti-tumor efficacy and facilitating tumor proliferation (74). Furthermore, the precursor tryptophan is converted into kynurenine in the body through indoleamine 2,3-dioxygenase, which demonstrates neuroactive characteristics and contributes to immunological control (75). Dopamine, a monoamine neurotransmitter in the CNS, impacts connections between the CNS and the immune system, while also mediating its involvement in cancer progression through the regulation of immunological responses (76). Multiple immune cells exhibit dopamine receptors (DR), with D1R and D5R associating with Gs proteins, whereas D2R, D3R, and D4R associate with Gi proteins. In patients with lung cancer, plasma dopamine levels may rise to five times the normal concentration; at this level, dopamine suppresses T cell proliferation and cytotoxicity via D1 receptors (46). Dopamine inhibits osteosarcoma cell proliferation by downregulating the ERK1/2 and PI3K/AKT pathways, and it may diminish the migration and invasion capacities of gastric cancer cells by obstructing the EGFR-AKT pathway (77).

### 3.3 Neural remodeling

Cancer not only causes neuronal remodeling but may also result in functional abnormalities in the nervous system (4). Patients with pancreatic cancer demonstrate notable neurological changes, including a decrease in sympathetic or adrenergic nerve fibers, but parasympathetic fibers remain comparatively stable (78). The phenomenon of “neural remodeling” is characterized by increased levels of neuroepithelial stem cell markers, such as nestin, and diminished expression of the glial transcription factor SOX10, suggesting the possible recruitment of neural progenitor cells to the pancreatic TME and dedifferentiation of peripheral glial cells (79). In the CNS, gliomas disrupt neural circuitry by promoting aberrant synaptogenesis, enhancing neuronal excitability, and triggering seizures. Pathological cerebral activity amplifies activity-dependent signaling pathways, promoting glioma proliferation. Cancers external to the CNS can also influence brain function from a distance, leading to complications such as sleep problems (80).

Inflammation in the TME promotes neuronal plasticity and the proliferation of nerve fibers, intensifying pain-related neural activity (81). This neuronal activity enhances vascular permeability in the tumor area and facilitates cancer-related pain via the development of abnormal neuromas. Macrophages serve a dual function in this process; M2 macrophages facilitate nerve healing and augment tumor innervation, but M1 macrophages are prone to inflicting nerve injury (38). Additionally, macrophages regulate angiogenesis through the secretion of VEGF, while Schwann cells utilize this mechanism to promote nerve regeneration. Tumor-associated macrophages express Sema4D, facilitating neurite development and contributing to tumor innervation (72). The effects of cancer and its therapies on the neurological system are under heightened scrutiny (82). Radiotherapy and chemotherapy seek to manage tumor proliferation; nevertheless, they may also disturb brain homeostasis, resulting in cognitive deficits (83). Damage to white matter and loss in hippocampus volume caused by these therapies correlate with cognitive impairments, including diminished attention and memory difficulties. In reaction to these detrimental effects, researchers are formulating techniques to enhance brain stem cell regeneration, with the objective of mitigating cognitive impairments associated with cancer therapy.

Cancer significantly impacts the nervous system. Breast cancer influences sleep patterns and metabolism by targeting particular neuronal populations (68). The bidirectional relationship between cancer and the neurological system creates a detrimental cycle, highlighting a crucial domain for future research in cancer therapy. In gliomas, tumors release synaptogenic substances that augment neuronal excitability and facilitate functional reorganization. Intraoperative electrophysiological investigations indicate that language function circuits are altered in glioma patients, with the synaptogenic factor TSP-1 produced by glioma cells likely serving as a critical mechanism for this alteration (84). Moreover, heightened neuronal excitability related to gliomas is intimately connected to seizures, and this aberrant neural activity intensifies the tumor-promoting effects of gliomas (85). Thus, the functional modification and usurpation of brain pathways that facilitate cognitive function represent a pivotal mechanism driving glioma advancement.

### 3.4 Regulation of the TME

Cancer creates a microenvironment that facilitates its growth and spread, aided by the nervous system and hindered by the immune system’s repression (86). Neural cells react to immunological signaling molecules, whereas immune cells respond to neurotransmitters and neuromodulators, significantly altering the activities of both systems in pancreatic cancer, breast cancer, melanoma, and ovarian cancer, etc. (87). Paracrine signals from nerves to tumor or stromal cells inside the TME modulate tumor growth and invasion, while substances originating from tumors alter adjacent nerves, facilitating more neural extension into the tumor environment (88). Neurogenic factors, including neurotransmitters and neuropeptides, regulate the movement and activity of immune cells; hence, changes in immune function can influence anti-tumor immunity and promote tumor-associated inflammation (89).

The systemic interactions between the nervous system and cancer can be facilitated by circulating paracrine signals, such as catecholamines, which directly communicate with tumor cells or other cell types within the TME (90). Consequently, tumor cells can affect the neurological system via circulating substances, thereby modulating the systemic neuro-cancer interaction, including the activity of the HPA axis (23). A complicated triangle interaction arises among neurons, immune cells, and cancer cells, involving the influence of the nervous system on the tumor immune milieu, tumor-promoting immunity, anti-tumor immunity, and associated mechanisms of immunotherapy. The brain system inhibits immune cell function by releasing neurotransmitters and neuropeptides (e.g., GABA), therefore diminishing their assault on cancer cells (70). This inhibitory effect transcends local tumor immune responses, influencing systemic immunological status and allowing cancer cells to flourish and multiply in a comparatively “secure” environment. The neural innervation of solid tumors can generate T cell exhaustion through sensory neurons, whereas the inhibition of CGRP can mitigate this T cell depletion (91). Optogenetic research has demonstrated that activating dopaminergic pathways from the ventral tegmental area (VTA) to the medial prefrontal cortex might alleviate the adverse effects of stress on tumor proliferation in breast cancer models (92). Moreover, VTA stimulation modifies the functional characteristics of MDSCs via influencing sympathetic innervation of the bone marrow, thus diminishing tumor growth in melanoma and lung cancer models.

The CNS has diverse functions in tumor immune evasion, both by diminishing immune responses through metabolites and by altering the tumor immunological microenvironment via neurotransmitters (93). In the TME, neurogenic stimuli stimulate receptors on cancer cells, triggering signaling cascades including PI3K/AKT, Ras/ERK, and PLCγ/PKC, which enhance cancer cell survival and proliferation in pancreatic cancer (94). TrkB expression is markedly increased in ovarian cancer cells, and HGF can stimulate TrkB expression (95). The BDNF/TrkB axis suppresses anchorage-independent apoptosis in ovarian cancer cells through the PI3K/AKT pathway, resulting in the development of drug-resistant cells. Substance P (SP) facilitates local endothelium-dependent vasodilation and tumor advancement by activating NK1 receptors associated with Gq and Gs pathways (96). Afferent nerve terminals and mast cells facilitate local vasodilation and inflammation via SP/CGRP and histamine, establishing a communication network among afferent nerve fibers, mast cells, and blood vessels, hence intensifying the TME. Substance P stimulates mast cells to secrete histamine, promoting vasodilation via G protein-coupled receptors in vascular smooth muscle cells. Mast cells facilitate the production and release of VEGF via IgE-mediated signaling pathways (IgE/FcϵRI/Fyn complex), hence enhancing tumor angiogenesis (97). Targeting these pathways may impede tumor growth by obstructing vasodilation and neovascularization.

In breast cancer, concentrations of the neuronal metabolite N-acetylaspartate (NAA) and its producing enzyme NAT8L are markedly increased. NAT8L reduces the creation of immunological synapses (IS), hindering the cytotoxic actions of NK cells and T cells, thereby obstructing the immune system’s assault on malignancies (3). Catecholamines, another category of neurogenic regulatory substances, concurrently influence immunological responses within the TME. Catecholamines promote macrophage polarization to the M2 phenotype and enhance regulatory T cell numbers, transforming the TME into an immunosuppressive state (98). Moreover, adrenergic signaling modulates T cell entry and exit via ADRB2; inhibiting ADRB2 amplifies the cytotoxic effects and migratory abilities of CD8^+^ T cells, therefore inhibiting tumor proliferation (99).

### 3.5 The impact of neuro-immune interactions on cancer therapy

A strong bidirectional relationship exists between the nervous and immune systems, where neurotransmitters and neuromodulators directly affect the activity, differentiation, and migration of immune cells, while alterations in the immune system also provide feedback to the CNS through neuro-immune regulatory pathways (100). This dynamic relationship presents innovative therapeutic targets for addressing tumor growth and introduces new avenues for cancer treatment. The neurological system’s function in modulating immune responses during infections and inflammatory reactions has been thoroughly investigated, and the reciprocal regulation mechanisms are also crucial (101). Mediators secreted by leukocytes, such as substance P, can facilitate tumor proliferation. In medication development, in addition to the approved neuromodulatory medicines in neurology, psychiatry, and internal medicine, there should be a proactive investigation of molecules with immunomodulatory properties (102). High-throughput screening, bioinformatics analysis, and preclinical validation facilitate the rapid identification of prospective therapeutic candidates targeting both the neurological and immune systems.

In light of advancing knowledge of neuro-immune-cancer interactions, therapeutic techniques should transition from single-target methodologies to multi-target, multi-pathway approaches in drug development (103). Combining neurotransmitter modulators with immune checkpoint inhibitors may enhance anti-tumor efficacy and diminish adverse effects. This integrated therapeutic approach has potential to become a vital avenue in future cancer treatment. Furthermore, neuro-immune interaction uncovers novel possibilities in targeted therapy, chemotherapy, and radiotherapy. Inhibiting particular neurological pathways, including ADRB2 signaling of the sympathetic nervous system, might augment the cytotoxicity of CD8^+^ T lymphocytes, therefore impeding the proliferation of malignancies like breast cancer (38). The modulation of cholinergic neurotransmission, which affects the activity of immunosuppressive T cells and macrophages within the spleen, exhibits potential for anti-tumor applications (104). Although interference with the normal processes of the CNS and PNS may restrict drug dosages, optimizing the dosage and creating personalized treatment regimens might enhance therapeutic effects while preserving the patient’s quality of life (65). Furthermore, promoting interdisciplinary collaboration and increasing the number of clinical trials will yield essential data regarding the safety and efficacy of these medications.

## 4 Molecular mechanisms of CNS-immune system interactions

### 4.1 CNS modulation of the immune microenvironment in cancer progression

Immune checkpoint inhibitors have established themselves as a conventional therapy for multiple cancer types. Investigations into neuro-immune signaling pathways have demonstrated that the nervous system can modulate the function of immune cells located in tissues and lymph nodes via several mechanisms (88). Integrating neuroactive pharmaceuticals with immune checkpoint inhibitors, cytotoxic treatments, or cancer vaccines may alter the immuno-tumor microenvironment, hence augmenting the anti-cancer efficacy (105). Neural signaling modulates the magnitude and duration of immunological responses by governing the movement and function of T cells (26). Lymph nodes possess abundant adrenergic and sensory nerve innervation, with β-adrenergic receptor signaling regulating T cell discharge from lymph nodes (4). Consequently, the modulation of brain signals can profoundly influence the retention and discharge of T cells from lymph nodes, thereby augmenting both innate immune responses and the anti-cancer effects caused by immune checkpoint inhibitors.

Neuropeptides and neurotransmitters are essential in modulating the tumor immunological microenvironment (100). The neuropeptide CGRP has been shown to induce CD8^+^ T cell exhaustion inside the melanoma microenvironment, thereby diminishing anti-tumor immune responses and facilitating melanoma growth. The link between the neuro-immune systems indicates that modulating particular neuropeptide or neurotransmitter signaling pathways could improve the efficacy of immunotherapy. The neurotransmitter GABA plays key functions in several malignancies (71). Primary tumors have demonstrated the ability to release neurotrophic factors or axon-guiding molecules, so attracting nerve innervation. Healthy breast tissue is abundantly supplied with sensory nerves, and the pathogenic presence of nerves in breast tumors correlates with adverse patient outcomes, indicating that nerve distribution may influence tumor spread. Studies indicate that breast cancer cells can provoke spontaneous calcium activation in sensory neurons, resulting in the production of the neuropeptide substance P (106). The study utilized 3D co-culture models of breast tumor cells and neurons, along with *in vivo* mouse models, to demonstrate that neuronal substance P enhances breast tumor development, infiltration, and metastasis. Patients with tumors with elevated substance P levels demonstrate markedly increased lymph node metastases. Substance P interacts with the tachykinin receptor TACR1 on tumor cells, resulting in the demise of a specific subset of cancer cells that exhibit high TACR1 expression. Apoptotic cells emit single-stranded RNA, which stimulates Toll-like receptor 7 (TLR7) in adjacent tumor cells, initiating a non-canonical gene expression program that facilitates metastasis. Utilizing the TACR1 antagonist aprepitant, a medication employed to mitigate nausea and vomiting induced by chemotherapy and surgery, has demonstrated efficacy in inhibiting the growth and metastasis of various murine breast cancer cell types by targeting this neuro-tumor signaling pathway.

Furthermore, malignant cells can assimilate into brain circuits via authentic neuron-glioma connections. Malignant glioma cells are interconnected via gap junctions, facilitating the propagation of neuron activity-dependent electrical currents throughout a densely linked neuro-glioma network (101). Post-synaptic electrical impulses facilitate cancer propagation by depolarizing the membrane potential of glioma cells, however the voltage-sensitive mechanisms involved are still to be clarified. The pro-carcinogenic effects of excitatory neurotransmission also encompass brain metastasis. Breast cancer cells that metastasis to the brain increase the expression of neurotransmitter receptors and elongate post-synaptic processes to capture neuron activity-dependent neurotransmitter signals (104). These signals activate receptor-mediated signaling cascades, resulting in inward currents in malignant cells and promoting the proliferation of brain metastases originating from breast cancer.

### 4.2 Key molecules and signaling pathways

Neural innervation plays a crucial role in the genesis and advancement of many cancers through sophisticated molecular pathways. Research has shown that neural innervation is essential in malignancies like gliomas, prostate cancer, pancreatic cancer, and gastric cancer (100, 101, 103, 104). Neurotransmitters such as glutamate, norepinephrine, and acetylcholine modulate tumor cell proliferation and migration by attaching to their specific receptors. Growth factors, such as nerve growth factor (NGF), brain-derived neurotrophic factor (BDNF), and glial cell line-derived neurotrophic factor, significantly impact the TME (40, 107). In high-grade gliomas, soluble neuroligin-3 (NLGN3) secreted by neurons and oligodendrocyte precursor cells interacts with receptors on tumor cell surfaces, activating the PI3K-mTOR signaling pathway that governs cell proliferation and survival, thus facilitating tumor growth and metastasis (108).

Alongside secreted neurotransmitters and growth factors, direct physical connections—tumor-nerve synapses—may develop between neurons and tumor cells. These synapses enable signal transmission between neurons and tumor cells, improving neural control of the tumor (66, 88). These connections not only deliver growth signals to the tumor but may also function as essential regulatory nodes within the TME, affecting tumor aggressiveness and therapy responsiveness. The transforming growth factor-β (TGF-β) signaling pathway is regarded as a crucial mechanism in the maturation of microglia and the maintenance of their homeostasis (45). TGF-β regulates microglial growth and function through many pathways, limiting overactivation and maintaining immunological homeostasis in the CNS, therefore reducing the risk of neuroinflammation-related injury. TGF-β may also contribute to the inhibition of tumor growth by regulating the inflammatory response of microglia (109). The CSF1R signaling pathway is another essential mechanism governing immunological responses in the CNS. CSF1R regulates the development and maintenance of microglial activity while also modulating their interaction with external signals, hence affecting immune responses within the TME and thereby influencing tumor progression (109).

### 4.3 Patterns of intercellular communication

The interplay between the CNS and the immune system relies on complex systems of intercellular communication, mostly mediated by neurotransmitters, cytokines, chemokines, and their corresponding receptors. The spleen, thymus, and lymph nodes, which are immune organs, get innervation from the sympathetic nervous system, and lymphocytes, a kind of immune cell, express adrenergic receptors (16). Furthermore, immune cells possess receptors for substances modulated by the neuroendocrine system, such as corticotropin-releasing hormone and glucocorticoids. Neuroregulatory processes can influence immunological activity, as specific brain regions modulate immune function through neural pathways, allowing behavioral and environmental factors, such as stress, to impact the immune system (110). Neuroanatomical studies indicate that the sympathetic nervous system extensively innervates all lymphoid organs, forming direct connections with T cells and plasma cells, referred to as “neuroeffector junctions.” Initially, the importance of parasympathetic modulation was questioned; however, subsequent research revealed an autonomous non-neuronal cholinergic system in lymphocytes that synthesizes acetylcholine through autocrine or paracrine signaling to regulate immune responses (63). T cells release acetylcholine upon activation, acting as autocrine or paracrine substances to modulate immune responses. The CNS regulates immune function through neurotransmitters and hormones, while the brain-immune axis enables behavioral and environmental factors, including stress, to influence immunological responses.

The immune system is regulated by the CNS and can also influence brain activity and behavior through signals such as pro-inflammatory cytokines (16). Immunological cells identify signals of infection or injury and transmit this information to the CNS, triggering immunological responses such as fever. Hypothalamic neurons modulate fever responses, which are associated with the activation of glucocorticoids and sympathetic nerves, hence affecting immunological function (65). Adrenergic receptor expression exhibits notable differences between innate and adaptive immune cells. Innate immune cells predominantly express α2, β1, and β2 receptors, while adaptive immune cells show a preferential expression of β2 receptors. The activation of these receptors plays a crucial role in modulating T and B cell responses through cAMP-dependent signaling pathways, which involve PKA and exchange proteins directly activated by cAMP. This signaling mechanism specifically enhances immunoglobulin (Ig) synthesis via the CREB signaling pathway (111). Research indicates that β2 receptor activation modifies IgG1 and IgE responses in B cells and affects inflammatory responses via the p38 MAPK signaling pathway (112).

Paracrine signaling plays a critical role in neuron-mediated regulation of glioma growth. Research indicates that insulin-like growth factor-1, as a paracrine factor regulated by neuronal activity, directly promotes glioma growth in the olfactory bulb region (77). This mechanism highlights the regional and circuit-specific interaction patterns between neurons and gliomas. Given the complexity and diversity of neurons, such circuits and neuron subtype-specific mechanisms are not uncommon in glioma development. In the intricate neuron-immune-cancer cell network, the multifaceted effects of paracrine signals become even more pronounced (113). For instance, midkine, released by retinal ganglion cells, stimulates CD8^+^ lymphocytes to secrete the chemokine CCL4, which subsequently activates glioma-associated microglia or macrophages to secrete CCL5. This signaling cascade ultimately affects NF1-associated low-grade glioma cells in the optic pathway, promoting their survival and progression (114). This underscores the pivotal role of the nervous system in shaping the tumor immune microenvironment and reveals its dual role in regulating tumor inflammation and anti-cancer immune responses. Notably, BDNF also plays a crucial role in neuron-glioma interactions (40). BDNF not only regulates the proliferation of healthy OPCs but also modulates the intensity of glutamatergic currents within glioma cells, reflecting the dynamic interplay between neurons and tumor cells (95). Furthermore, the synaptic adhesion molecule NLGN3, another key paracrine factor, is critical for glioma progression. The absence of NLGN3 significantly impedes the progression of both high- and low-grade gliomas (108). NLGN3 promotes glioma cell proliferation by activating multiple oncogenic signaling pathways, including PI3K-mTOR, SRC, and RAS, offering a novel perspective on the integration of gliomas with neural circuits.

Signaling across the blood-brain barrier (BBB) represents one of the key mechanisms of interaction between the CNS and the immune system, particularly under pathological conditions such as cancer (115). When the CNS is exposed to infection, injury, or tumor stimuli, it rapidly initiates defense mechanisms by releasing cytokines (e.g., IL-1β, TNF-α) and chemokines (e.g., CXCL10, CCL2). These signaling molecules act both locally and remotely by recruiting peripheral immune cells to cross the BBB. In response to inflammatory stimuli, adhesion molecules such as ICAM-1 and VCAM-1 are upregulated in BBB endothelial cells (116). Typically expressed at low levels under normal conditions, these adhesion molecules bind integrins like LFA-1 on immune cells, forming a “molecular bridge” that enhances interactions between immune cells and endothelial cells, facilitating immune cell migration across the BBB (117). Guided by chemokines, immune cells approach endothelial cells and cross the BBB via restructuring of tight junction proteins. This process involves MMPs and nitric oxide, which mediate dynamic adjustments in endothelial cell junctions, widening the spaces between cells to allow immune cell passage. Immune cells cross the BBB through two main routes: paracellular and transcellular pathways (118). In the paracellular pathway, immune cells migrate through gaps between endothelial cells, while in the transcellular pathway, immune cells are engulfed by endothelial cells through endocytosis and released into the CNS via exocytosis. This mechanism is particularly effective for certain immune cell types, such as T cells and macrophages. Once immune cells have crossed the BBB and entered the CNS, they continue to migrate within the CNS along chemokine gradients and other signaling molecules to precisely locate infection or injury sites. In the CNS, immune cells can directly kill cancer cells and regulate local immune responses by releasing cytokines, promoting inflammation control, assisting in tissue repair, and exerting anti-tumor effects within the TME (66). Additionally, the activity of immune cells is modulated by neurotransmitters within the CNS, such as norepinephrine and GABA, which influence immune cell function through neuro-immune interactions, further regulating anti-tumor immune responses.

### 4.4 Cytokines and chemokines

In pathological circumstances like cancer and inflammation, cytokines (e.g., TNF-α and IL-1β) engage with the endothelial cells of the BBB, resulting in a marked elevation of adhesion molecules such as ICAM-1 and VCAM-1 (116). The increase of these adhesion molecules is essential for establishing molecular connections that enable immune cells to attach to endothelial cells, therefore promoting their retention on the BBB surface in anticipation of crossing. Simultaneously, chemokines (e.g., CCL2, CXCL12) establish concentration gradients along the endothelial surface of the blood-brain barrier, drawing immune cells that express the relevant receptors (e.g., CCR2, CXCR4) toward the barrier. These gradients accurately lead immune cells across the blood-brain barrier and towards areas of inflammation or malignancies. Immune cells, when nearing the BBB, utilize surface integrins (e.g., LFA-1) to preferentially attach to the increased adhesion molecules (e.g., ICAM-1, VCAM-1) present on endothelial cells, therefore establishing robust cell adhesions (117). This facilitates the retention of immune cells on the blood-brain barrier surface, establishing a basis for eventual translocation. During inflammatory conditions, immune cells traverse the BBB via two primary pathways. The initial pathway is the paracellular route, wherein immune cells “squeeze” through intercellular gaps between endothelial cells, which are temporarily expanded by the activity of MMPs and other regulatory factors, involving cytoskeletal reorganization and modification of tight junction proteins (119). The second is the transcellular route, where certain immune cells enter endothelial cells through an endocytosis mechanism and are subsequently released into the CNS via exocytosis.

Chemokines facilitate immune cell migration and recruit bone marrow-derived immune cells, including M2-type macrophages, via interactions with particular receptors including CCR2 and CXCR4 (120). These cells are involved in modulating the CNS milieu, either facilitating anticancer activity or engaging in immune regulation. Prostaglandin E2 might enhance the immuno-suppressive activity of MDSCs, which impede immune responses in the TME by obstructing the development of normal immune cells, thus sustaining localized immune suppression (121). Moreover, G-protein-coupled receptors (GPCRs) are essential in the signaling mechanisms of chemokines and cytokines. These GPCRs govern immune cell motility, adhesion, and trans-BBB movement via downstream signaling pathways, hence influencing immune control within the TME (122). In the setting of tumors, the GPCR signaling system amalgamates signals from cytokines, adhesion molecules, and chemokines, facilitating immune cell infiltration via the blood-brain barrier, thereby altering the tumor immunological milieu within the CNS and affecting tumor development and prognosis.

### 4.5 Neurotrophic factors and immunoregulatory molecules

Cancer cells can release neurotrophic substances, including NGF and Artemin, to stimulate the development of nerve fibers toward tumor tissues (123). These factors not only encourage the formation of nerve fibers but also directly influence cancer cells by activating surface receptors, so enhancing their proliferation and survival. For instance, NGF can stimulate signaling pathways in cancer cells, so exacerbating their malignancy. Moreover, these neurotrophic factors alter the TME, enhancing tumor cell adaptability and invasiveness, hence promoting a more aggressive phenotype. Neural invasion is a prevalent occurrence in several malignancies, especially in pancreatic cancer (124). Pancreatic cancer cells demonstrate a pronounced attraction to neurons, proliferating along nerve fibers, which facilitates cancer invasion and dissemination. In contrast, neural invasion is less significant in colorectal cancer, possibly owing to diminished response to neurotrophic agents.

During neural invasion, nerves infiltrated by cancer cells display increased concentrations of neurotrophic factors and chemokines, fostering a more conducive microenvironment for the migration and invasion of cancer cells towards the nerves (107). Furthermore, subsequent to infiltrating the nerves, cancer cells enhance the production of specific cytoskeletal elements, including kinesin family member 14 and Rho GDP dissociation inhibitor 2, which are essential for cellular motility and invasion. Research indicates that the axon-guidance molecule SLIT2 functions as an inhibitor of neural invasion in cancer (125). SLIT2 is aberrantly expressed in human pancreatic cancer and highly metastatic breast cancer, and it has been demonstrated to inhibit neural invasion. In breast cancer models, SLIT2 expression is substantially elevated in the endothelial cells of highly metastatic breast cancer, and its elimination dramatically diminishes tumor innervation. This indicates that SLIT2 may have a significant inhibitory function in modulating the TME, especially for neural innervation and invasion. Neurotrophic factors and immunoregulatory substances are deeply engaged in the intricate connections between cancer cells and the neurological system throughout cancer progression. These variables critically influence neural invasion and cancer dissemination by controlling nerve fiber growth, cancer cell survival and proliferation, and alterations in the TME. The identification of inhibitory molecules such as SLIT2 offers novel targets for comprehending and addressing neuronal invasion in cancer.

## 5 Therapeutic strategies targeting neuro-immune interactions

**Figure 3:**
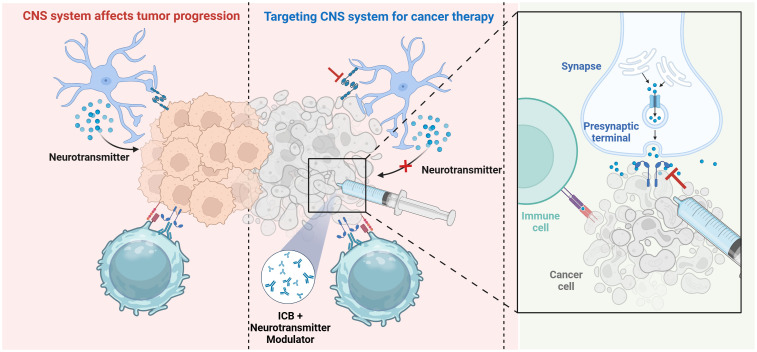
Therapeutic strategies targeting neuro-immune interactions. Therapeutic approaches focusing on neuro-immune cell interactions, including immune checkpoint inhibitors, alongside innovative technology such as brain-computer interfaces and nanodelivery systems, demonstrate potential in enhancing treatment efficacy. Created with BioRender.com.

The CNS is crucial in tumor development and immune control, as its interactions with the immune system markedly affect cancer progression and therapy efficacy (126). Given this intricate system, researchers have investigated methods to regulate neurotransmitters, the neuro-immune axis, and suppress tumor angiogenesis to disrupt cancer progression. Neurotransmitter modulators have been shown to enhance the TME by promoting immune cell activation and migration, hence directly suppressing tumor growth (127). Furthermore, these medicines can mitigate neurogenic inflammation and related discomfort, enhancing patients’ quality of life. CNS-targeted medicines, particularly those aimed at specific gene mutations or inhibiting brain signaling pathways, have markedly prolonged patient survival, notably in the management of intricate CNS malignancies such as glioblastoma. Therapeutic techniques aimed at the CNS-cancer interaction are becoming vital complements to conventional treatments, such as surgery, radiation, chemotherapy, and advanced immunotherapy (16). Targeting neuro-cancer interactions alone may be insufficient for the entire eradication of tumors; nevertheless, neuro-regulatory medicines could be integral to effective treatment protocols for specific refractory cancers, including high-grade gliomas and pancreatic cancer. Disrupting neuro-cancer connections can decelerate tumor growth and augment cancer cell susceptibility to chemotherapy and radiotherapy. In the future, neuro-regulatory medicines are expected to be combined with traditional cancer treatments and immunotherapies, creating individualized and holistic therapy approaches, especially for tumor types influenced by neuro-cancer interactions. These combination medicines not only enhance clinical results but also provide novel approaches for addressing resistant tumors (**Figure 3**). Additionally, advanced delivery systems, such as nanotechnology-based platforms, are facilitating the precise delivery/of neuro-regulatory medicines across the blood-brain barrier, maximizing therapeutic efficacy while minimizing systemic side effects. These strategies are particularly impactful in the treatment of complex CNS malignancies like glioblastoma, where conventional therapies often fail.

### 5.1 Potential therapeutic targets

The intricate interplay between the brain system and immune cells reveals several potential targets for cancer treatment. These interactions elucidate the essential function of the nervous system in regulating the tumor immune milieu and may substantially influence the management of resistant malignancies (16). Investigating the interaction between the neurological system and cancer has potential to enhance conventional treatment methods. Nf1 mutations augment the baseline action potential firing in neurons of optic nerve tumor mice, resulting in the synthesis of midkinase that influences T cells and induces the secretion of CCL4, which subsequently activates microglial cells to release CCL5, a crucial mitogen for glioma cell proliferation (114). Nf1 mutations cause baseline hyperexcitability via the regulatory mechanism of HCN1 channels, and the antiepileptic medication lamotrigine can target this channel, normalizing midkinase expression and reducing glioma proliferation. Sema4F is elevated in prostate cancer, and its inhibition can diminish tumor neurogenesis (128). Furthermore, Sema4F is involved in the interaction between cancer cells and fibroblasts, promoting the migration of cancer cells along fibers. The NMDAR is an essential excitatory neurotransmitter receptor in the CNS, demonstrating aberrant expression in several malignancies (39). Potential targets for modulating neuro-immune cell interactions are summarized in **Table 1**.

**Table 1:** Potential targets for tumors via neuro-immune targeting.

Marker	Cancer types	Substrate/Target	Mechanism	Reference
NF1	Optic nerve glioma	CCL4/CCL5	Increased baseline action potential firing of neurons in optic glioma mice, leading to the production of midkine	(113)
Sema4F	Prostate cancer	Plexin B1/2	Promotes cancer cell proliferation and migration, facilitating cancer cell movement along fibers	(128)
NMDAR	Glioma, pancreatic cancer, breast cancer	mTOR,ERK	Inhibits cancer cell proliferation	(129)
SLIT2	Breast cancer, pancreatic cancer	ROBO1	Regulates the TME, particularly neural innervation and invasion	(125)
TLR7	Breast cancer	TACR1	Activates pro-metastatic gene expression	(106)
NAT8L	Breast cancer	N-acetylaspartate	Disrupts the formation of the immunological synapse, impairing the cytotoxic functions of NK cells and T cells	(3)
CSF1	CSF1R	**/**	Affects immune responses within the TME	(84)
ADRB2	Breast cancer	**/**	Regulates the cytotoxic function and migratory capacity of CD8^+^ T cells	(130)
NLGN3	Glioma	PI3K-mTOR	Regulates cell proliferation and survival, promoting metastasis	(131)
NK1	Melanoma; Glioblastoma	Substance P	The neurokinin-1 receptor binds to the neurotransmitter substance P, promoting tumor cell growth and metastasis	(132)
NGF	Pancreatic cancer	ATG7	Promotes the growth of nerve fibers	(107)

### 5.2 Potential therapeutic drugs and clinical trials

Recent research has revealed how the complex relationships between the neurological and immune systems affect tumor development and treatment responses. Recent research has revealed various small molecule medicines, especially those aimed at the CNS, that can influence immune cells within the TME, consequently hindering cancer progression and metastasis (**Table 2**) (112, 126, 133). Targeting specific cytokines produced by tumor cells (e.g., IL-6, TNF-α) to modulate immune cells in the TME has shown potential efficacy in clinical trials. Additionally, researchers are formulating innovative anti-tumor pharmaceuticals that specifically target inflammatory signaling pathways in neurons, including the NF-κB and JAK/STAT pathways. These medicines not only alleviate tumor-associated inflammation but may also augment the immune system’s capacity to identify and eradicate tumor cells. Integrating pharmacological agents that address neuro-immune interactions with conventional immune checkpoint inhibitors may have synergistic benefits, enhancing patient outcomes. Research indicates that the integration of PD-1/PD-L1 inhibitors with neuromodulators markedly improves tumor management in murine models. In conclusion, therapeutic approaches aimed at neuro-immune cell interactions exhibit significant potential, and ongoing clinical trials yield essential data to facilitate future advancements in this domain (**Table 3**). As our comprehension of these intricate mechanisms advances, it is anticipated that novel pharmaceuticals and therapeutic procedures may arise, providing renewed hope for cancer patients.

**Table 2:** Small molecule drugs targeting the neuro-immune axis for cancer treatment.

Drug	Substrate/Target	Mechanism	Research stage	Indications	Reference
Propranolol	β2AR	Inhibits tumor cell-induced nerve growth factor production	On the market	Breast cancer, ovarian cancer	(36)
Serotonin, norepinephrine	5-HT1A	Inhibits the growth and spread of cancer cells	On the market	Breast cancer, glioma	(134)
Memantine	N-methyl-D-aspartate receptor	Reduces neuronal hyperexcitability while also inhibiting tumor growth and metastasis	On the market	Brain metastases	(135)
GW2580	CSF1R	Inhibits the migration of tumor-induced intraneural macrophages	In preclinical	Glioblastoma	(109)
larotrectinib,entrectinib	TrkA, TrkB, TrkC	Blocks Trk signaling	In preclinical research	Neuroblastoma	(136)
Trifluoperazines	ERK, AKT	Reduces the proliferation and invasion of cancer stem cells	In preclinical research	Lung cancer; Breast cancer	(76, 137)
Brigatinib	ALK	Inhibits cancer cell proliferation	On the market	Astrocytoma, glioblastoma	(138)
Tovorafenib	RAF	Inhibits the signals of tumor growth and cancer cell survival.	On the market	Glioma; Metastatic melanoma	(139)
Perampanel	AMPA	Inhibits cancer cell proliferation	On the market	Glioma	(140)
Aprepitant	TACR1	Inhibits the binding of substance P to the TACR1 receptor	On the market	Breast cancer	(106)

**Table 3:** Potential new drugs targeting the neuro-immune axis for the treatment of human cancers approved by the FDA.

Drug	Mechanism	Cancer type	Status	Intervention/treatment	Clinical trial
EEDVsMit	Anthracycline with engeneic delivery vehicle	Recurrent/refractory solid or CNS tumors	Phase 2	Intravenous injection	NCT02687386
Lutathera	Somatostatin analogue	High-grade CNS tumors	Phase 1-2	Intravenous injection	NCT05278208
Dacomitinib	EGFR tyrosine kinase inhibitors	Advanced EGFR-mutant NSCLC	Phase 2	Oral	NCT04675008
Midazolam	Benzodiazepines	Unresectable or metastatic gastrointestinal stromal tumors and other advanced solid tumors	Phase 2	Oral	NCT04908176
Riluzole	GABA uptake inhibitor	Breast Cancer	Phase 1	Oral	NCT02796755
Cabazitaxel	Chemical semi synthetic small molecule compounds of taxane class	Refractory solid tumors	Phase 1-2	Intravenous injection	NCT01751308
Eribulin mesylate	Macrolides	Recurrent or refractory solid tumors	Phase 1	Intravenous injection	NCT02171260
TKI258	Multi target receptor tyrosine kinase inhibitors	Advanced solid tumors	Phase 1	Oral	NCT01596647
AZD1775	Wee1 inhibitor	Advanced solid tumors	Phase 1b	Oral	NCT02341456
Dexanabinol	Cannabinoid derivatives	Advanced solid tumors	Phase 1	Intravenous injection	NCT01489826
SAR302503	JAK2 inhibitor	Refractory solid tumors	Phase 1	Oral	NCT01585623
Ridaforolimus	mTOR inhibitor	Advanced solid tumors	Phase 1	Intravenous injection	NCT00704054
Ramucirumab	VEGFR2 antagonist	Refractory solid tumors	Phase 1	Intravenous injection	NCT02564198
AZD1775	Wee1 inhibitor	Refractory solid tumors	Phase 1	Oral	NCT03333824

### 5.3 Traditional Chinese medicine

Increasing evidence indicates that traditional Chinese medicine possesses anti-tumor properties, particularly in enhancing anti-tumor immunity (141). The desiccated aerial components of Eupatorium lindleyanum DC. are extensively employed for its anti-inflammatory and immunomodulatory properties. The principal active molecule, eupatorium lactone B, has been identified to directly engage with the USP7 protein via molecular probe technology. It covalently alters USP7, inducing conformational modifications that activate the Keap1/Nrf2 signaling pathway, consequently reducing neuroinflammation and offering neuroprotection (142). Emodin demonstrates considerable effectiveness in treating multiple sclerosis, especially through the modulation of the PI3K-Akt signaling pathway. Emodin tightly interacts with PI3K, AKT1, and NFKB1, thereby downregulating the activation of pertinent signaling pathways in microglia in the experimental autoimmune encephalomyelitis (EAE) model. It inhibits the phosphorylation of PI3K, Akt, NF-κB, and Myd88 in both M1 and M2 microglia, decreasing the levels of CD86 and CD206 markers, therefore mitigating inflammation and offering neuroprotective properties (143). Astragalus Polysaccharides facilitate the differentiation of neural stem cells (NSCs) into oligodendrocytes, hence improving myelin regeneration and altering the ecological niche of NSCs by diminishing CD8^+^ T cell infiltration. The immunomodulatory effect transpires via diminished IFN-γ release by CD8^+^ T cells, promoting NSC differentiation into oligodendrocytes and subsequently mitigating neurological impairments. This mechanism exhibits considerable efficacy in the EAE model (144). Gastrodin activates the Nrf2 signaling pathway in microglia, inducing a neuroprotective phenotype and alleviating chronic neuroinflammation. This phenotypic shift markedly enhances depressed and anxious behaviors in experimental mice (145). Lycium barbarum glycopeptide demonstrates efficacy in a neuromyelitis optica spectrum disorder model by mitigating neuroinflammatory lesions and enhancing neurological function via the suppression of NF-kB signaling activation in microglia and astrocytes (146).

### 5.4 Applications of new technologies

The existence of the blood-brain barrier constrains the capacity of numerous pharmaceuticals to penetrate the CNS, consequently diminishing the effectiveness of targeted cancer immunotherapy in this area (147). In recent years, various innovative technologies have been investigated to improve the efficacy of cancer treatment and substantially mitigate the difficulties related to targeting the CNS. Brain-computer interfaces (BCIs) have transformative potential for cancer treatment (148). The essence of BCI technology is its ability to monitor cerebral activity in real time and control neural signal transmission via a closed-loop feedback system (149). On one side, BCIs can accurately detect abnormal neural processes in the brain, such as tumor-associated signals, and affect the progression of cancer within the CNS by altering the intensity or patterns of these signals. Conversely, BCIs can augment immune responses by modulating the interactions between the brain and the immune system, thus enhancing the effectiveness of targeted cancer therapy. BCI technology can precisely locate tumor-associated neural activity in brain tumor treatment and employ closed-loop feedback systems to modulate neural signals, thereby preventing tumor growth. Applications encompass the insertion of intracortical depth electrodes or subdural electrodes to record deep brain activity, facilitating the location and monitoring of malignancies (150). BCIs can monitor chemotherapy-induced peripheral neuropathy, enabling early detection of lesions and mitigating patient symptoms through the modulation of neural signal intensity. Furthermore, closed-loop BCIs utilize integrated algorithms to react instantaneously to variations in brain signals, thereby dynamically refining treatment approaches and augmenting immune responses against cancer cells via the manipulation of neuro-immune signaling pathways (65).

By integrating nanomedicine with bioelectronics, BCI systems that utilize wireless-powered luminescent devices and near-infrared technology can accurately activate nanoparticles in the TME through modulation of wavelength and light intensity, resulting in localized tumor heating and effective inhibition of tumor growth (141). This method not only circumvents the intrusive techniques linked to conventional photothermal therapy but also exhibits sustained therapeutic efficacy in freely roaming murine mice. This technique can alter CNS-immune interactions. BCI technology can remotely control photothermal therapy and accurately target nanoparticles to regulate neuro-immune signaling pathways, thereby augmenting immune responses against cancer cells, marking a significant advancement in the treatment of refractory brain tumors like glioblastoma (151). Nanomaterial technology demonstrates significant potential in traversing the blood-brain barrier, safeguarding therapeutic molecules from degradation, and precisely targeting the brain (118). In recent years, artificial nanoparticles and nanovaccine technologies have shown fresh promise for cancer therapy. These methods augment vaccine delivery effectiveness by enhancing blood-brain barrier permeability and exploiting compromised lymphatic drainage in glioblastoma multiforme. Furthermore, tailored nanoparticles provide the continuous and regulated release of antigens or adjuvants and exhibit precise targeting abilities for endothelial cells within the blood-brain barrier, including applications in RNA-based vaccine administration. In GBM treatment, drug delivery systems include multiple routes, such as systemic administration, local delivery (e.g., subdural and intraparenchymal administration), and intranasal delivery (115). Different routes of administration significantly impact the biodistribution of nanoparticles and drug accumulation in the brain. For instance, intranasal delivery, as a non-invasive method, can bypass the BBB via the olfactory nerve, enhancing drug delivery efficiency. Research indicates that drug accumulation in the brain from intranasal delivery is significantly higher than that from intravenous administration, with reduced distribution to non-target organs.

**Figure 4:**
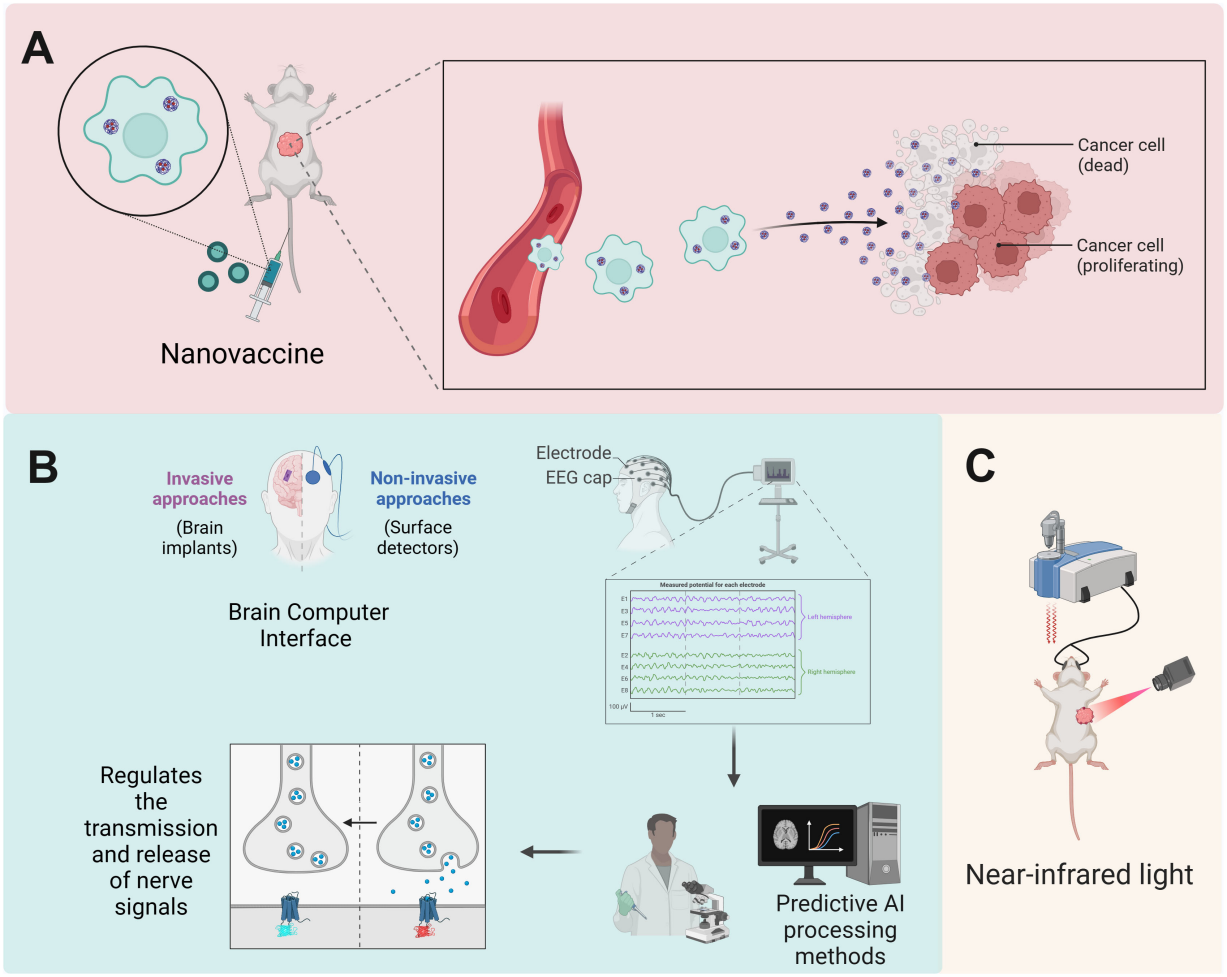
Emerging technologies targeting CNS-immune cell interactions promise to optimize therapeutic outcomes. **(A)** Innovative nanovaccines, including STING agonists and mRNA formulations, stimulate robust innate and adaptive immune responses, promoting the infiltration of effector immune cells and offering therapeutic promise in cancer immunotherapy. **(B)** BCIs facilitate real-time monitoring of cerebral activity and modulation of neural signal transmission, enabling the detection of tumor-associated signals and enhancement of immune responses against cancer through neuro-immune signaling pathways. **(C)** By integrating nanomedicine with bioelectronics, BCIs employing wireless-powered luminescent devices enable precise activation of nanoparticles in the tumor microenvironment, resulting in localized heating and effective tumor growth inhibition. Created with BioRender.com.

Nanovaccine technologies, including as STING agonists, mRNA vaccines, and neoantigen nanovaccines, are becoming focal points in cancer immunotherapy research. These vaccines stimulate both innate and adaptive immunological responses, specifically via the STING signaling pathway, promoting the infiltration of CD8^+^ T cells, NK cells, and dendritic cells, thereby demonstrating therapeutic promise (152). For instance, STING-activating nanovaccines produced from human heavy chain ferritin nanoparticles have exhibited substantial immune activation effects in murine cancer models. Nonetheless, nanovaccines encounter obstacles in clinical applications, such as the synthesis and functionalization of nanomaterials, efficacy in antigen presentation, and the need to navigate the intricate immunosuppressive milieu of tumors. Creating nanoparticles that specifically target immune cells necessitates meticulous attention to material composition, surface modification, particle size, shape, and surface chemical characteristics. The route of administration significantly influences the dispersion and overall effectiveness of nanomaterials. Future research must optimize the design and functionalization of nanomaterials, incorporating novel nanovaccine technologies to improve the efficacy of cancer immunotherapy. These novel technologies offer prospective solutions to challenges such as quick degradation, abbreviated half-lives, and elevated costs linked to conventional vaccinations, thereby enhancing therapy alternatives for cancer patients and maybe increasing survival rates (**Figure 4**). Toxoplasma gondii, a parasite adept at traversing the blood-brain barrier, has recently been employed for protein drug delivery (153). This research employed genetic engineering to leverage the rod-shaped morphology and dense granule protein secretion mechanism of Toxoplasma for the effective delivery of therapeutic proteins, including MeCP2, into brain cells. This technology surpasses the current size constraints of viral vectors, presenting an innovative strategy for addressing neurological illnesses.

## 6 Challenges and future perspectives

Although the brain’s crucial function in the immune system is acknowledged, the precise regulation processes associated with cancer remain intricate and obscure. The relationship between cancer and the neurological system is complex, involving numerous signaling molecules and pathways. A primary issue in contemporary research is converting these intricate systems into practical applications. Can the tumor-neural axis function as a precise target to produce extensive clinical advantages? Currently, targeted medicines aimed at this axis remain in the exploratory phase. Moreover, the potential for brain modulation to synergize with established treatments—such as immunotherapy, radiation, and chemotherapy—and to be effectively incorporated into current therapeutic protocols constitutes a significant challenge. Ultimately, accurately identifying patients who are most likely to benefit from these therapeutic options by histopathological or other diagnostic tools necessitates further research. Despite the development of various peptides, neoantigens, cellular, and mRNA vaccines, the advancement of these therapies in clinical trials has been sluggish due to the immunosuppressive TME, the existence of the blood-brain barrier, and the intrinsic instability of the vaccines, resulting in only marginal enhancements in efficacy.

As our comprehension of the processes governing the interactions between cancer and the neurological system advances, forthcoming research will investigate several new domains and trajectories. The processes of interaction at the cellular level require more elucidation, especially about how cancer cells specifically modulate neural activities and the consequences of this modulation for cancer progression. Furthermore, comprehending the impact of the nervous system on the TME—particularly its modulation of the TME—will be a crucial focus for future research. Furthermore, the advancement of innovative therapeutic approaches centered on the interplay between the neurological system and cancer will be a primary emphasis of forthcoming research. This includes many methods such as pharmacological treatments, genetic therapies, immunological therapies, and biotechnology interventions. Researchers must investigate how to properly utilize these treatment methods to address the particular interactions between cancer and the neurological system. Interdisciplinary collaboration is essential for investigating the relationships between cancer and the neurological system. The amalgamation of insights and methodologies from neurology, cancer, and pharmacology can yield a more holistic understanding of the intricate mechanisms governing these interactions. Insights from neuroscience about neural signaling and neuronal function can assist oncologists in comprehending the interactions between cancer cells and the nervous system. Simultaneously, progress in pharmacology can provide essential technological assistance for the development of innovative therapeutic techniques. The impact of brain control on tumor progression necessitates more examination. Investigations into denervation in malignant tumors have uncovered the crucial function of nerves in tumor biology, since they can promote tumor cell proliferation and spread via the TME. Additional research is required to ascertain if additional malignancies, such as bladder and kidney tumors, demonstrate peripheral nerve invasion and whether they are influenced by neural control, particularly within the CNS. Furthermore, considering the antagonistic link between the sympathetic and parasympathetic nervous systems within the cardiovascular system, it is pertinent to explore if this antagonism is also present in their regulation of malignancies.

The improved comprehension of neuro-tumor interactions is elucidating the clinical potential of neuromodulatory treatments. Interferon therapy demonstrates some success in cancer treatment; nonetheless, its detrimental effects on the neurological system, including psychiatric or emotional problems like sadness and anxiety, provide considerable challenges. In extreme instances, individuals may have hallucinations or exhibit suicidal inclinations. The neurotoxicity of interferon-α is frequently associated with dosage, and the occurrence of neuropsychiatric adverse responses is linked to the patient’s psychiatric history, family history, treatment dosage, and duration. Consequently, before commencing interferon therapy, healthcare providers must thoroughly assess the patient’s psychiatric history and consult with mental health professionals as needed to reduce the likelihood of negative neurological consequences during treatment. Precision medicine and individualized treatment strategies are set to assume a crucial role in future clinical practice. Customizing treatment protocols to the unique neuro-immune interaction profiles of individual patients will emerge as a crucial focus in cancer therapy. By focusing on the interplay between the nervous system and malignancies, tailored therapeutic solutions can improve treatment efficacy while reducing superfluous side effects, thereby offering patients superior therapy alternatives.
